# Etching-free pixel definition in InGaN green micro-LEDs

**DOI:** 10.1038/s41377-024-01465-7

**Published:** 2024-05-24

**Authors:** Zhiyuan Liu, Yi Lu, Haicheng Cao, Glen Isaac Maciel Garcia, Tingang Liu, Xiao Tang, Na Xiao, Raul Aguileta Vazquez, Mingtao Nong, Xiaohang Li

**Affiliations:** Advanced Semiconductor Laboratory, Electrical and Computer Engineering Program, CEMSE Division, King Abdullah University of Science and Technology (KAUST), Thuwal, 23955-6900 Kingdom of Saudi Arabia

**Keywords:** Inorganic LEDs, Displays, Optoelectronic devices and components

## Abstract

The traditional plasma etching process for defining micro-LED pixels could lead to significant sidewall damage. Defects near sidewall regions act as non-radiative recombination centers and paths for current leakage, significantly deteriorating device performance. In this study, we demonstrated a novel selective thermal oxidation (STO) method that allowed pixel definition without undergoing plasma damage and subsequent dielectric passivation. Thermal annealing in ambient air oxidized and reshaped the LED structure, such as *p*-layers and InGaN/GaN multiple quantum wells. Simultaneously, the pixel areas beneath the pre-deposited SiO_2_ layer were selectively and effectively protected. It was demonstrated that prolonged thermal annealing time enhanced the insulating properties of the oxide, significantly reducing LED leakage current. Furthermore, applying a thicker SiO_2_ protective layer minimized device resistance and boosted device efficiency effectively. Utilizing the STO method, InGaN green micro-LED arrays with 50-, 30-, and 10-µm pixel sizes were manufactured and characterized. The results indicated that after 4 h of air annealing and with a 3.5-μm SiO_2_ protective layer, the 10-µm pixel array exhibited leakage currents density 1.2 × 10^−6^ A/cm^2^ at −10 V voltage and a peak on-wafer external quantum efficiency of ~6.48%. This work suggests that the STO method could become an effective approach for future micro-LED manufacturing to mitigate adverse LED efficiency size effects due to the plasma etching and improve device efficiency. Micro-LEDs fabricated through the STO method can be applied to micro-displays, visible light communication, and optical interconnect-based memories. Almost planar pixel geometry will provide more possibilities for the monolithic integration of driving circuits with micro-LEDs. Moreover, the STO method is not limited to micro-LED fabrication and can be extended to design other III-nitride devices, such as photodetectors, laser diodes, high-electron-mobility transistors, and Schottky barrier diodes.

## Abstract (web summary)

The selective thermal oxidation method was used to define high-performance micro-LED pixels without suffering plasma etching and sidewall damage.

## Graphical abstract

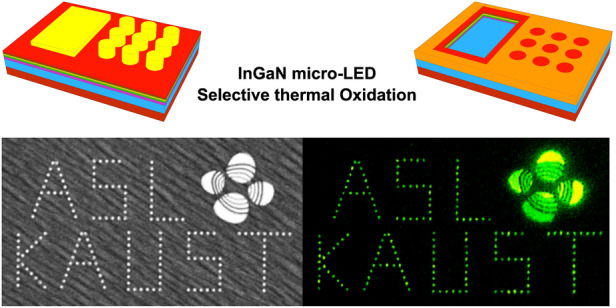

## Introduction

Recently, InGaN-based blue, green, and red micro-LEDs have garnered significant attention and interest due to their exceptional features such as high contrast, intense brightness, excellent energy efficiency, and long device lifetimes, positioning them as strong contenders as the next-generation display technology^1–3^. Compared to large-scale LEDs, micro-LEDs exhibit superior current spreading and heat dissipation capabilities, which contribute to enhanced device performance^4,5^. Furthermore, the sustainable high current density of micro-LEDs also holds promise for applications in high-bandwidth visible light communication^6,7^. However, to define micro-LED pixels, plasma or ion etching is widely adopted to selectively remove portions of the active region, resulting in severe sidewall damage and defect formation^8^. These defects act as non-radiative recombination centers and paths for leakage current, hampering the efficiency of micro-LEDs^9–11^. The negative impact from plasma damage is not limited to the sidewall surface but, depending on etching conditions, penetrates to a certain depth, known as the “dead zone.” Due to a higher area ratio between the sidewall and mesa top surface, sidewall damage has a more pronounced impact on the efficiency of smaller devices, known as the efficiency size effect or sidewall effect in micro-LEDs^12,13^. Several mainstream methods have been reported to mitigate this issue:

(i) Remove the sidewall damage regions. Etchants such as KOH and TMAH were extensively employed to selectively etch the damaged regions at the sidewall facet, reducing micro-LED leakage current^14,15^. (ii) Surface passivation of sidewalls: material surfaces are at the end of the periodic lattice, aiding in increasing the number of dangling bonds. At the same time, the sidewall surface also bears a significant amount of plasma damage^16^. Therefore, surface passivation is an effective method to passivate dangling bonds and suppress surface states caused by ions and plasmas. Recently, ALD dielectrics like Al_2_O_3_ have been widely used to deposit on micro-LED sidewalls for surface passivation^17,18^. ALD-AlN has also been demonstrated as an efficient passivation material for III-nitride-based micro-LEDs^19^. Additionally, various approaches, such as rapid thermal annealing to repair sidewall defects and (NH_4_)_2_S treatment to remove unstable native oxides and form monolayer sulfide passivation, have been introduced to optimize micro-LEDs^20,21^. (iii) Modulate the current path to steer it away from the sidewall regions in order to reduce non-radiative carrier recombination near the mesa edge. For instance, people have proposed an oxide-confined structure to reduce current diffusion to the sidewall^22^. Hang and Zhang et al. employed the resistive ITO/*p*-GaN junction and Ta_2_O_5_ high-k insulator to modulate the band structure and reduce hole concentration near damaged regions^23,24^. Kirilenko et al. utilized H_2_ plasma to passivate Mg acceptors of *p*-type layers near the sidewall and formed an insulating region to suppress carrier non-radiative recombination^25^.

However, these methods all involve subsequent optimization following plasma etching for pixel definition. Avoiding the use of plasma etching may provide an alternative solution for the development of micro-LEDs, which warrants further investigation. In this study, we proposed an innovative selective thermal oxidation (STO) method that achieved pixel definition without plasma damage and dielectric passivation, potentially avoiding the harmful effects brought about by ion and plasma etching. Thermal annealing in ambient air oxidized the LED layers and formed insulating oxides. However, LED pixels were protected by a SiO_2_ layer to prevent oxidation and maintain their luminescent functionality. As critical design parameters, annealing time and SiO_2_ thickness exhibited significance in micro-LED performance and were systematically investigated in this work. Using the STO method, we demonstrated micro-LED arrays with pixel sizes as small as 10-µm and provided a comprehensive performance analysis for devices with different pixel sizes.

## Results

### Device fabrication process

**Fig. 1:**
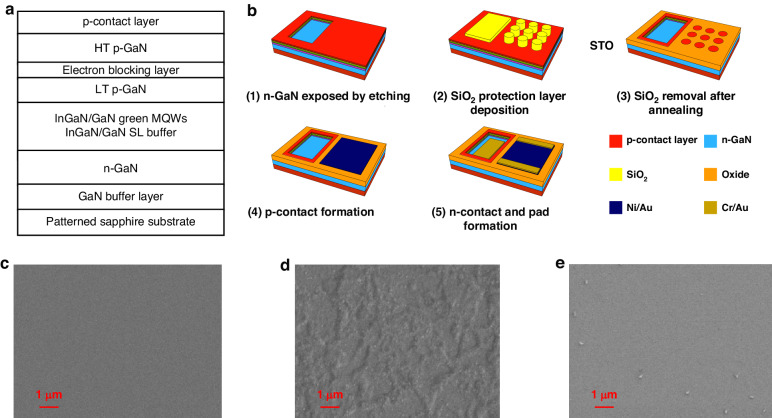
**Device fabrication method.** **a** Schematic structure of the InGaN-based green LED wafer. **b** Fabrication process flow of micro-LED arrays fabricated using selective thermal oxidization (STO). SEM images of the LED surface, **c** reference sample without thermal oxidization, **d** 4-h thermal oxidization without SiO_2_ protection, and **e** 8-h thermal oxidization with 3.5-µm SiO_2_ protection and SiO_2_ removal by HF vapor

The LED fabrication process began with InGaN-based green LED wafers, as schematically depicted in Fig. 1a, grown on the sapphire substrate. These wafers included the GaN buffer layer, *n*-GaN electron injection layer, two pairs InGaN/GaN superlattice buffer layer (3/17 nm), eight pairs InGaN/GaN multiple quantum wells (MQW) (4/15 nm) with photoluminescence wavelength around 551 nm, 50 nm LT *p*-GaN, 25 nm electron-blocking layer, 125 nm HT *p*-GaN and a *p*-contact layer. Figure 1b illustrates the STO process flow. Initially, a plasma etching process (BCl_3_, Cl_2_, and Ar plasma) was employed to expose the *n*-GaN layer, facilitating subsequent *n*-contact formation. It’s worth noting that the etching process was not for the pixel definition, and the etched regions were far away from the pixels, thus causing no damage to them. SiO_2_ was then deposited using plasma-enhanced chemical vapor deposition and patterned by dry etching (C_4_F_8_ and O_2_ plasma with Cr as a hard mask) as a protective layer on the surfaces of *n*-GaN and *p*-contact layer. The areas covered with SiO_2_ on *p*-contact layer served as the pixel region. In this study, micro-LED pixels of 50-, 30-, and 10-µm sizes were designed, with the total emitting area being held constant (0.0177 mm^2^). Subsequently, the LED wafers were annealed in ambient air in the tube furnace to selectively oxidize the portions of the regions not protected by SiO_2_. Simultaneously, the dense structure of SiO_2_ blocked the penetration of oxygen from the air, thereby protecting the underlying LED structure^26,27^. Similar to the thermal oxidation process of Si, the SiO_2_ surface layer functions as a barrier to oxygen diffusion, shielding the underlying Si from additional oxidation^28^. This was also the so-called STO step mentioned in this work. Figure 1c–e shows scanning electron microscope (SEM) images of the LED surfaces: (a) the reference sample without thermal oxidation, (b) no SiO_2_ protection with 4 h of thermal oxidation, and (c) 8 h of thermal oxidation with 3.5-µm SiO_2_ protection, followed by SiO_2_ removal using HF vapor. The results indicated that the LED surface without SiO_2_ protection became extremely rough compared to the reference sample due to the occurrence of oxidation. In contrast, with 3.5-µm SiO_2_ protection, the LED surface remained smooth even after 8 h of thermal annealing in the ambient air, indicating the importance of the SiO_2_ layer in blocking oxygen during the STO process.

**Fig. 2:**
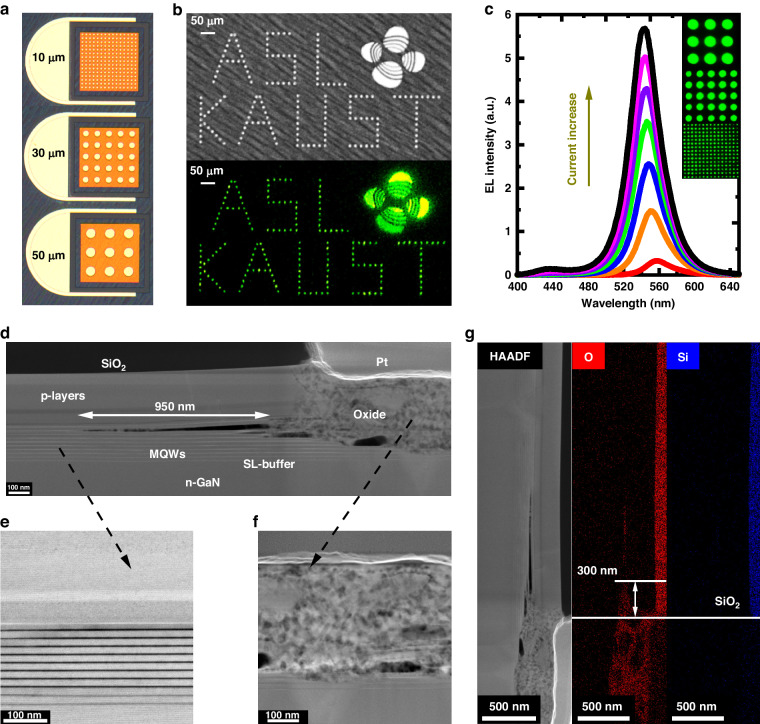
**Demonstration of green micro-LED arrays**. **a** Optical images of micro-LED arrays with 10-, 30-, and 50-µm pixels. **b** Device optical image and luminescent image of a 10-µm pixel display schematic diagram with ITO transparent contact. **c** Emission spectrum under various currents (the insets are some top-emission devices by STO process with 10-, 30-, and 50-µm pixels for illustration). **d** Cross-section TEM image at oxide/LED interface with 4 h annealing. **e** Magnified TEM image of the LED MQW structure under SiO_2_ protection. **f** Magnified TEM image of the oxidized material without SiO_2_ protection. **g** EDX element (Si and O) distribution

The annealing temperature is crucial for thermal oxidation performance and needs to be carefully considered. Given the stability of the InGaN MQWs, especially the indium diffusion issue, the annealing temperature should not be excessively high, even with SiO_2_ protection^29,30^. On the other hand, the temperature should be sufficiently high to oxidize GaN and InGaN in the absence of SiO_2_ protection. Therefore, a final annealing temperature of 900 °C was chosen for this work, as it has been demonstrated to be a relatively lower temperature for thorough oxidation of GaN films^31^. The heating and cooling rates were set at 5 °C/min during the thermal oxidization. The stability (no peel-off) of the SiO_2_ mask under prolonged high-temperature annealing is crucial for achieving STO. Indeed, we have observed in a very small number of devices the disappearance of the SiO_2_ mask after annealing. However, this phenomenon is extremely rare in the devices we have fabricated. In Supplementary Material SM1, we presented optical and SEM images of pixels before annealing, after annealing, and after removing SiO_2_. We speculate that the peeling off of SiO_2_ may depend on the surface cleanliness of the wafer and the deposition method and material quality of SiO_2_. These factors may potentially affect the adhesion between SiO_2_ and the LED surface at high temperatures. Following annealing, SiO_2_ was removed by HF vapor at a substrate temperature of 40 °C. It was found that HF vapor exhibited crucial etching selectivity between SiO_2_ and other oxides^32^. Hence, we opted for HF vapor to minimize the thickness decrease of formed oxide materials when removing the SiO_2_ protective layer. The high etch selectivity of HF vapor toward SiO_2_ and the formed oxides has been demonstrated in Supplementary Material SM2. After the removal of SiO_2_, cathodoluminescence (CL) could be observed in pixels, forming a strong contrast with the oxidized regions, which are shown in Supplementary Material SM3. Finally, 20/300 nm Ni (e-beam deposition)/Au (sputter deposition) (annealed in O_2_ at 550 °C for 300 s) and 30/200 nm Cr/Au (sputter deposition and annealed in N_2_ at 300 °C for 300 s) were deposited as the *p*- and *n*-electrodes, respectively^33^. Since the formed oxides already served as insulation between pixels, there was no need for additional dielectric deposition in the process. Simultaneously, procedures like selective dielectric etching required in traditional micro-LED fabrication for subsequent metal contact and the associated photolithography alignment were no longer necessary. Therefore, the proposed STO process for micro-LED fabrication is a self-aligned technique, reducing the complexity of photolithography for small-size device fabrication. The wafer size used in the experiment was 1 cm × 1 cm. After mounting the chips on sapphire substrates and wire bonding, the optical characteristics of micro-LEDs were on-wafer measured, and the light was extracted from the bottom of the chip. Through the aforementioned process, 50-, 30-, and 10-µm pixel micro-LED arrays have been fabricated, and device optical images are shown in Fig. 2a. To demonstrate the feasibility of achieving pixel clarity through the STO method, the device optical image and luminescent image of a 10-µm pixel display schematic diagram with ITO transparent contact is presented in Fig. 2b. Figure 2c provides the corresponding emission spectrum under different injection currents.

In Fig. 2d–g, we examined transmission electron microscopy (TEM) images near the oxide/LED interface after 4 h of STO. As expected, the TEM images showed a clear boundary between the SiO_2_-protected and SiO_2_-unprotected regions in Fig. 2d. Under SiO_2_ protection, the LED structure remained intact, exhibiting a clear superlattice structure (enlarged in Fig. 2e) and excellent crystalline quality in the MQW (please see in Supplementary Material SM4). However, once the SiO_2_ protection was lost, most of the *p*-layer and MQW were oxidized (enlarged in Fig. 2f). The oxidized layers show a distinctly polycrystalline nature with multiple crystal orientations mixed together (please see in Supplementary Material SM4). Furthermore, we found a 950 nm long crack near the oxide/LED interface in Fig. 2a. It was likely that the crack resulted from the thermal mismatch between the oxide and nitride materials during the heating and cooling stages of the STO process^34^. It has also been found in some other pixels we characterized shown in Supplementary Material SM5, indicating this phenomenon is not an isolated case. We believe that if such cracks were present in smaller-sized devices, they would cause significant damage to device yield and performance. However, for the micro-LEDs in this study, which had dimensions of 10-µm or larger, the negative impact of the crack was considered to be limited because the pixel size was much larger than the crack width. Furthermore, since the crack occurred at the top of the MQW, it may help prevent hole injection into the active region near the oxide/LED interface, reducing non-radiative recombination and improving micro-LED efficiency. However, this suspicion requires further systematic experiment and characterization support. Figure 2g presents the energy-dispersive X-ray (EDX) spectroscopy element distribution. The results clearly demonstrated the significant presence of oxygen elements in areas lacking SiO_2_ protection, implying structural oxidation. Besides, we confirmed that the lateral diffusion distance of oxygen in the protected region was ~300 nm after 4-h thermal oxidization.

**Fig. 3:**
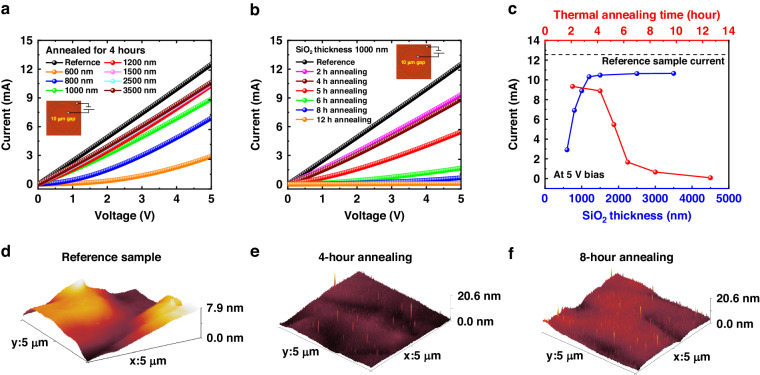
Effect of thermal annealing on *p*-layer currents. **a** The *p*-layer current with various SiO_2_ protection layer thicknesses annealed for 4 h. **b** The *p*-layer current with different annealing times with 1000 nm SiO_2_ protection. **c** A summary of *p*-layer current at 5 V bias with various SiO_2_ thicknesses and annealing times. 3D AFM surface image of (**d**) reference sample without thermal oxidization, **e** 4-h thermal oxidization with 3.5-µm SiO_2_ protection and SiO_2_ removed by HF vapor, and **f** 8-h thermal oxidization with 3.5-µm SiO_2_ protection and SiO_2_ removed by HF vapor

After thermal annealing in ambient air, the nitrides without SiO_2_ protection underwent oxidation, forming oxides. The insulating properties of these oxides were crucial for reducing LED leakage current and enhancing device efficiency. Wang et al. reported the formation of Ga_2_O_3_ through the oxidation of *p*-type GaN and its application in deep ultraviolet photodetectors. As the annealing time initially increased, the film resistance rapidly increased. However, with prolonged annealing time, the resistance of the Ga_2_O_3_ began to decrease^35^. Consequently, a sufficiently long and appropriate annealing duration was considered essential for improving the oxide insulation and reducing micro-LED leakage current in this work, as will be demonstrated in our subsequent device characterization. However, a longer annealing time may introduce some parasitic effects. (i) While the dense structure of SiO_2_ can effectively suppress oxygen from entering the pixel interior, it is challenging to achieve complete blocking. As the annealing time increases, more oxygen elements may enter the LED from the air. (ii) As previously reported in research, oxygen elements from SiO_2_ can diffuse into GaN under high-temperature thermal annealing. With an extended annealing time, the depth and concentration of oxygen diffusion significantly increase^36^. Since the LED wafers used in this study were based on InGaN and GaN epitaxial layers, this oxygen diffusion effect also occurred. On the one hand, infiltrating oxygen may disrupt the original LED structure, reduce current conduction, and affect luminescent functionality through oxidation. On the other hand, after thermal annealing, the *p*-side metal contacts may deteriorate due to the formation of surface oxides, and these surface oxides are complicated to eliminate by HF vapor etching. It is well known that the conductivity of the *p*-layer and the metal contact performance significantly determined the operation voltage of fabricated micro-LEDs at the same current density, which is, in other words, the device resistance. Using circular transmission line measurement patterns with 10-µm spacing shown in Fig. 3a, b, we investigated the influence of SiO_2_ thickness and annealing time on the current conduction of the *p*-type layer with Ni/Au contacts. The influence of SiO_2_ thickness and annealing time on the *p*-layer current at 5 V bias has also been summarized in Fig. 3c. In the experiment, SiO_2_ thickness was verified by the profile meter, and minor thickness variations from material deposition were considered to have no significant impact on our conclusions. Following thermal annealing, the SiO_2_ protective layer was removed by HF vapor. The reference sample with the same Ni/Au contact did not suffer any thermal annealing for comparison with other samples.

Figure 3a reveals that under the same 4-h annealing conditions, as the SiO_2_ protective layer thinned from 3500 to 600 nm, the behavior of the Ni/Au metal contacts gradually shifted from pure Ohmic to Schottky behavior. Simultaneously, the current in the *p*-type layer significantly decreased from 10.65 to 2.92 mA. Since the annealing time was the same for all samples, the diffusion of oxygen elements from SiO_2_ to the LED should be similar. Therefore, it proved that a thicker SiO_2_ layer may be more effective in preventing oxygen intrusion from the air. However, in Fig. 3b, we observed that under the same SiO_2_ thickness protection, the *p*-contact and *p*-type layer current conduction deteriorated with a longer annealing time, particularly beyond 4 h. The analysis suggested that even though a thicker SiO_2_ layer was beneficial in impeding oxygen diffusion from the air to the sample, it could not effectively prevent the penetration of oxygen elements from SiO_2_ into the LED. With prolonged annealing time at 900 °C, oxygen diffusing from SiO_2_ would oxidize the LED surface, impairing its conductivity and the performance of the metal contact. This also explains why, despite using a 3500 nm thick SiO_2_ protective layer, as shown in Fig. 3a, the current in the annealed samples decreased compared to the reference samples without thermal annealing. To further demonstrate the analysis above, in Fig. 3d–f, we compared the atomic force microscopy (AFM) surface morphologies of samples with 3.5-μm SiO_2_ protection and different annealing times (0, 4, and 8 h), measured by Bruker’s Dimension Icon. Compared to the non-annealed reference sample, samples annealed for 4 and 8 h still maintained a relatively smooth surface overall, consistent with the information in the previous SEM images. However, locally, we observed that the annealed samples exhibited numerous protrusions on the surface, and this phenomenon was slightly more pronounced in the sample annealed for 8 h (please see in Supplementary Material SM6). We believe this was due to oxygen diffusion from SiO_2_ into the LED, resulting in its slight oxidation of the surface and a minor increase in surface roughness.

In summary, it was essential to carefully control the annealing time and increase the SiO_2_ protection thickness to reduce leakage current while maintaining reasonable operation voltage or device resistance.

### Micro-LED array performance with different STO times

**Fig. 4:**
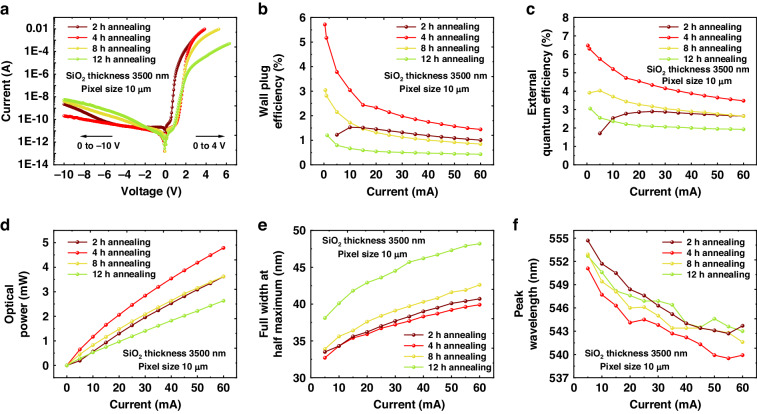
**Micro-LED array performance with different STO times.** **a** I–V characteristic, **b** wall-plug efficiency, **c** external quantum efficiency, **d** light output power, **e** spectrum FWHM, and **f** peak wavelength of devices A–D with 10-µm pixels and STO for 2, 4, 8, and 12 h. 1 mA corresponds to the current density of 5.6 A cm^−2^ for our micro-LED arrays

To explore the performance of micro-LEDs fabricated through the STO method, we designed four samples with STO durations of 2, 4, 8, and 12 h (referred to as devices A–D). The SiO_2_ protective layer thickness used in the devices was 3.5 μm. Figure 4a displays the I–V curves of the investigated devices. The results indicated that, compared to device A with a 2-h annealing, device B, annealed for 4 h, exhibited lower reverse leakage current at −10 V, 2.1 × 10^−10^ A (1.2 × 10^−6^ A/cm^2^), suggesting that properly increased annealing time helped improve oxide insulation and reduce the leakage current. Leakage behavior in device A was also observed at low forward voltages (around 0–1 V), which raised following the voltage much more rapidly than in device B due to additional defect-related current paths. However, with the extended annealing time in devices C and D, the reverse leakage current increases again to 10^−8^ to 10^−9^ A at −10 V, indicating that 4 h was the preferred annealing time for controlling leakage current compared to other annealing times.

**Fig. 5:**
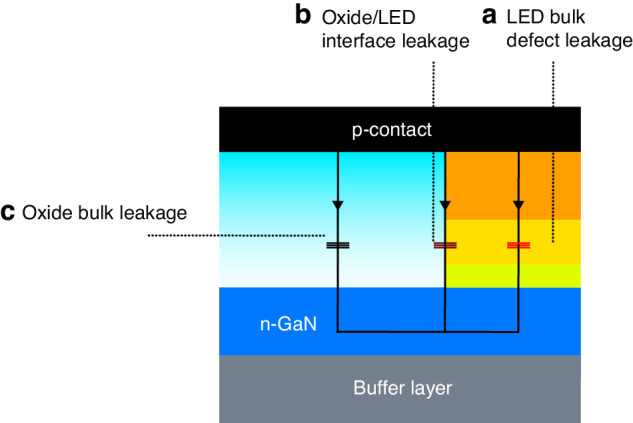
Leakage current path schematic diagram in the micro-LED fabricated by STO. **a** LED bulk defect leakage. **b** Oxide/LED interface leakage. **c** Oxide bulk leakage

Figure 5 illustrates all existing leakage current paths, including LED bulk leakage, oxide/LED interface leakage, and oxide bulk leakage. It should be noted that due to the diode I–V behavior, both LED bulk leakage and oxide/LED interface leakage significantly increased under forward voltage and LED emission. More current flowed through the defect regions under forward voltage, leading to significant non-radiative recombination and efficiency losses. It should be noted that the LED bulk defect is related to material epitaxy and is relatively independent of the fabrication process; therefore, it was not mainly considered in this work. As for the oxide bulk region, it was farther from the pixel, making it challenging for the current to spread to that area under forward voltage. It had high resistance (current < 10^−8^ A at −10 V), generating only negligible leakage current and non-radiative recombination during LED emission. From the I–V characteristics, device A exhibited a significant increase in current at low forward voltages, a phenomenon widely observed in micro-LEDs with severe sidewall defects^37^. Therefore, we suspected the oxide/LED interface leakage was considerable in device A and became a dominant factor for the leakage current. It also led to a more accelerated increase of the leakage current after a specific revere voltage (around −4 V). For devices C and D, unlike A, they exhibited a slower increase in leakage current at low forward voltages. We inferred that oxide/LED interface leakage in devices C and D was insignificant, and oxide bulk leakage was the main factor contributing to their reverse leakage. An oxidation time exceeding 4 h may lead to a decrease in the bulk insulation of the formed oxide, which aligned with the trend reported in ref. ^35^, and explains why devices C and D had higher reverse leakage currents compared to device B.

Consistent with the previous analysis, devices A and B, annealed for 2 and 4 h, respectively, exhibited similar forward operating voltages. A 3.5-µm SiO_2_ layer assisted in blocking oxygen from the environment, and the diffusion of oxygen elements in SiO_2_ did not significantly affect device resistance when the annealing time was shorter than 4 h. However, after annealing for more than 4 h, even with sufficiently thick SiO_2_, the device conductivity was greatly reduced. For annealing durations of 4, 8, and 12 h, the working voltage at 10 A/cm^2^ increased from 2.9 V to 3.75 and 8.0 V. We believe that oxygen diffused from SiO_2_ to the LED, thus leading to the surface oxidization, which was the primary reason for the device resistance increase. Based on the observed behavior, It was demonstrated that device B, with 4 h of STO, exhibited the best performance with low-leakage current and device resistance.

This conclusion was further validated through the on-wafer wall-plug efficiency (WPE) and external quantum efficiency (EQE) in Fig. 4b, c. For device A, the WPE and EQE peaks were observed at a higher current (1.53% at 10 mA) and (2.89% at 25 mA) compared to other devices, and the efficiency degradation was highly suppressed. This was a typical characteristic of high-leakage devices (LED bulk defect leakage and interface leakage) such as nanowire-LEDs and micro-LEDs^38,39^. Leakage paths contributed to more Shockley–Read–Hall recombination, equivalent to an increase in the “*A*” value (Shockley–Read recombination coefficient) in the internal quantum efficiency ABC model shown in Eq. 1, where other parameters include: “$\eta$” is the internal quantum efficiency, “${R}_{\rm{sp}}$” is the spontaneous emission rate, “${R}_{\rm{SRH}}$” is the Shockley–Read recombination rate, “${R}_{\rm{Auger}}$” is the Auger recombination rate, “$B$” is the radiative recombination coefficient, “${\rm{C}}$” is the Auger recombination coefficient, and “$n$” is the carrier concentration^40^.

1 $$\eta \left({IQE}\right)=\frac{{R}_{\rm{sp}}}{{R}_{\rm{SRH}}+{R}_{\rm{sp}}+{R}_{\rm{Auger}}}=\frac{B{n}^{2}}{{An}+{{Bn}}^{2}+{{Cn}}^{3}}$$

According to this equation, compared to low-leakage devices with a smaller “*A*” value, the efficiency peak in high-leakage devices appeared at a higher current density, and the efficiency drop is less pronounced^41^. However, the overall efficiency of such devices was poor. The efficiency curve of device A was highly consistent with the above feature and corroborated with the leakage behavior in the I–V characteristics presented earlier. In contrast to other devices, device B exhibited the highest WPE and EQE (5.72% and 6.48% at 0.4 mA) due to its low-leakage current and appropriate operating voltage. WPE and EQE were expected to be further improved by device packaging, optimizing wafer epitaxy, and optimizing STO conditions. In contrast to devices A and B, devices C and D, although experiencing a significant growth in reverse leakage after extended annealing, did not exhibit a significant current value increase at efficiency peak. Consistent with our previous analysis, the reverse leakage of devices C and D primarily came from the oxide bulk path, which did not significantly impact device performance. However, as mentioned earlier, prolonged annealing led to oxygen diffusion from SiO_2_ to the pixel, further resulting in surface oxidation. Therefore, the operating voltages and device resistance were higher for devices C and D. The WPE of devices C and D was only 3.04% at 0.6 mA and 1.20% at 1.3 mA, respectively. The large device resistance of devices C and D results in heat generation during device operation, which can also lead to the deterioration of EQE. Besides, as shown in Fig. 4d, the output power at 60 mA for devices A–D is 3.618, 4.787, 3.622, and 2.633 mW, respectively.

Furthermore, we measured the spectral full width at half maximum (FWHM) for devices A–D, as shown in Fig. 4e. Due to band-filling effects and heat generation, the FWHM of all devices increased with increasing current^42^. At a current of 60 mA, the FWHM values for devices A–D were 40.7, 39.9, 42.6, and 48.2 nm. The noticeable increase in FWHM for devices C and D compared to devices A and B was attributed to higher device resistance and increased heat generation. Moreover, as shown in Fig. 4f, the peak wavelength for devices A–D gradually decreased with increasing current due to carrier filling and polarization field screening^43,44^. However, we did not find significant evidence of wavelength variation with annealing time. The wavelength fluctuations between different devices due to the non-uniformity in epitaxial growth, but remained within a reasonable range. The similarity of wavelength across all four samples suggested that the quantum well structure was not significantly compromised under the protection of SiO_2_, and indium inside the MQW did not diffuse extensively. In the realm of quantum well stability, the influence of SiO_2_ remains ambiguous, prompting the need for continued and in-depth investigation in forthcoming research endeavors. The spectrum information of the measured devices can be found in Supplementary Material SM7.

### Size dependence of Micro-LED array performance with 4-h STO

**Fig. 6:**
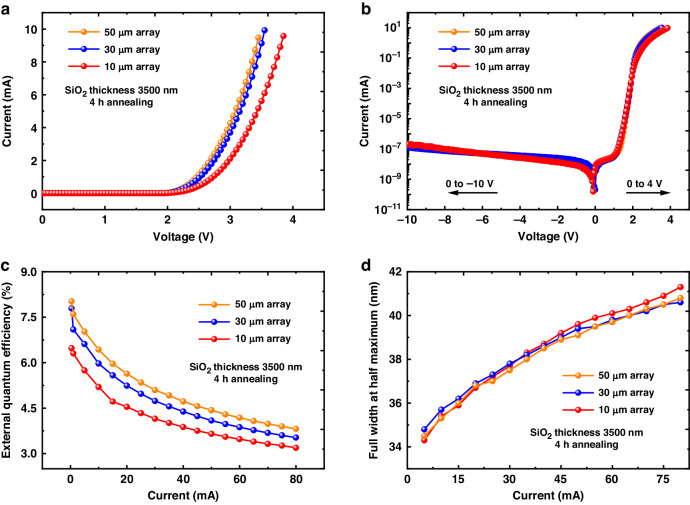
**Size dependence of micro-LED array performance.** **a** I–V characteristic at forward bias, **b** I–V characteristic in log scale, **c** external quantum efficiency, and **d** spectrum FWHM of devices with 10-, 30-, and 50-µm pixels for 4-h STO. Note: 1 mA corresponds to the current density of 5.6 A cm^−2^ for our micro-LED arrays

Furthermore, shown in Fig. 6a–d, we investigated the size dependence (50, 30, and 10 µm) of the I–V behavior, EQE, and spectral parameters for device B, which exhibited the highest efficiency among all sample annealed for various time. It was observed that smaller pixel arrays had slightly higher operating voltages at the same current, especially for the 10-µm micro-LED shown in Fig. 6a. We suspect some reasons about this phenomenon. First, it may be related to the HF vapor etching process. In larger pixel arrays, the material surface, after removing SiO_2_ by HF vapor, may have fewer residues and a cleaner surface. More reaction residues may lead to a higher operation voltage of the 10-µm device. Additionally, since the thermal oxidation process was isotropic, oxygen penetrated not only vertically but also laterally. Since the total pixel area was the same for all micro-arrays, smaller pixel arrays had relatively larger “side-surface” areas, indicating that more relative regions were exposed to the laterally diffused oxygen. This may also lead to an increase in operating voltage. Besides, the found cracks at the device edge may block carrier injection and worsen the device resistance. Under reverse bias, all devices exhibited stable and low-leakage current of about 1 × 10^−6^ A/cm^2^ at −10 V, as shown in Fig. 6b. Compared to micro-LEDs reported in other studies that utilized conventional plasma etching and dielectric passivation processes, this leakage current was at a relatively low level^45^. In conventional plasma-etched micro-LEDs, as the size decreases, leakage current rapidly increases due to sidewall defects caused by the plasma. However, with pixel definition using the STO method, the leakage current for the 10 µm array only increased slightly compared to the 30- and 50-µm arrays, indicating that the size effect on leakage current was suppressed.

Nevertheless, at 0.4 mA, on-wafer EQE decreased gradually from 8.02% to 7.79% and 6.48% as the pixel size changed from 50 to 30 and 10 µm, as shown in Fig. 6c. We believe that the efficiency degradation of smaller-sized devices was related to the leakage current and large resistance of the device. The latter led to a higher heat generation and junction temperature. Due to Auger recombination and carrier overflow, all devices exhibited similar efficiency droop at higher currents^46,47^. Further investigation of underlying mechanisms and process optimization, such as ambient control, annealing time, and subsequent fabrication processes, are necessary to enhance micro-LED efficiency, especially for small-sized devices. Finally, we measured the FWHM of the emission spectra shown in Fig. 6d. All devices exhibited similar FWHM. Compared to the 10 µm pixel device (41.3 nm), the 30 and 50 µm pixel devices had slightly lower FWHM at 80 mA (40.6 and 40.8 nm), possibly due to lower device resistance and less heat generation. All output power curves and spectrum information of the measured devices can be found in Supplementary Materials SM7 and SM8.

## Discussion

In conclusion, we have demonstrated pixel definition for micro-LEDs using an STO method without plasma damage and dielectric passivation. SiO_2_ thickness and annealing time were considered crucial factors in achieving excellent selectivity between oxidized and unoxidized regions. The oxidized regions exhibited satisfactory oxide insulation properties, while the unoxidized regions maintained their original structure and functionality, resisting the effects of thermal oxidation. This high contrast was crucial for achieving high-performance and small-sized micro-LEDs through STO. Through a series of studies, 10-µm pixel green micro-LED arrays with an on-wafer EQE of 6.48% have been realized using the STO method. Notably, the leakage current density was only slightly size-dependent and highly suppressed to 1.2 × 10^−6^ A/cm^2^ at −10 V in our 10 µm pixel array. Besides, the limited lateral oxygen diffusion (around 300 nm) beneath the SiO_2_ protection layer after 4 h annealing offered prospects for further reducing micro-LED dimensions. In order to achieve more applications based on micro-LEDs, such as tiny micro-displays for AR/VR, the size of micro-LEDs should be reduced to as small as 2 micrometers. In Supplementary Material SM9, we did preliminary demonstrations of the STO approach on 2.3-µm micro-LED pixilation. However, experiment conditions still need significant optimization to control oxidation and cracks to improve device performance. Besides, we have proposed some design strategies in Supplementary Material SM10 that could serve as references for the future fabrication of ultra-small micro-LEDs using the STO method. The achievable minimum pixel linewidth may be as small as 0.6 µm if considering a lateral oxidation distance of 300 nm. Furthermore, the main purpose of fabricating micro-LED arrays in this study is to measure the average performance of pixels. The same approach can be directly applied to individual micro-LED fabrication. The process flow is detailed in Supplementary Material SM11. Individual micro-LEDs fabricated through the STO method can be applied for display purposes through subsequent mass transfer processes such as stamp transfer and laser induced forward transfer. While further process optimization and a deeper understanding of the mechanisms involved are urgently needed to enhance micro-LED efficiency using STO manufacturing, we believe this research will provide valuable guidance for the future fabrication of micro-LEDs without ion and plasma damage. The physical mechanisms and device fabrication methods involved also promise to extend to other III-nitride electronic and optoelectronic devices in the future.

## Methods

The LED wafers were annealed in ambient air in the tube furnace OTF-1200X. DEKTAK XT profile meter was used to verify the SiO_2_ thickness. AFM surface morphologies were measured by Bruker’s Dimension Icon. SEM and CL images were collected by the Zeiss Merlin system. TEM images were measured by a Cs probe corrected microscope system from Thermo Fisher Scientific. Micro-LED I–V electrical performance was measured by the Keithley 4200 probe station system. Micro-LED optical performance was characterized by EVERFINE integration sphere system.

### Supplementary information

supplementary material
