# Supplementary material for: Etching-free pixel definition in InGaN green micro-LEDs
Source: Light Sci Appl. 2024 May 24;13:117. doi: 10.1038/s41377-024-01465-7 (PMC11116531; doi:10.1038/s41377-024-01465-7)
Supplement: Supplementary file 1 — supplementary material [file 41377_2024_1465_MOESM1_ESM.pdf]

Supplementary Material

Title: Etching-free pixel definition in InGaN green micro-LEDs

Zhiyuan Liu, Yi Lu, Haicheng Cao, Glen Isaac Maciel, Tingang Liu, Xiao Tang, Na Xiao, Raul Aguileta Vazquez, Mingtao Nong, and Xiaohang Li*

Section SM1:

The stability of the SiO_2 mask under prolonged high-temperature annealing is crucial for achieving STO. Indeed, we have observed in a very small number of devices the disappearance of the SiO_2 mask after annealing, as shown in Fig. S1a and Fig. S1b (n-electrode parts were not etched in these samples), leading to the loss of protection in the pixel area and subsequent oxidation. However, this phenomenon is extremely rare in the devices we have fabricated. We speculate that the peeling off of SiO_2 may depend on the surface cleanliness of the wafer and the deposition method and material quality of SiO_2.

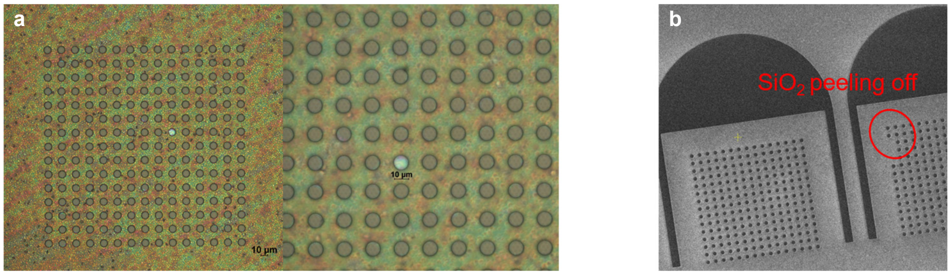

Fig. S1. (a) Optical microscopy and (b) SEM images of peeling off of SiO_2 after the STO process.

In Fig. S2a to Fig. S2c, we presented optical and SEM images (by Helios system) of pixels before annealing, after annealing, and after removing SiO_2 using HF vapor. The n-electrode regions were not etched in this illustration. No peeling off of SiO_2 was found in these good devices.

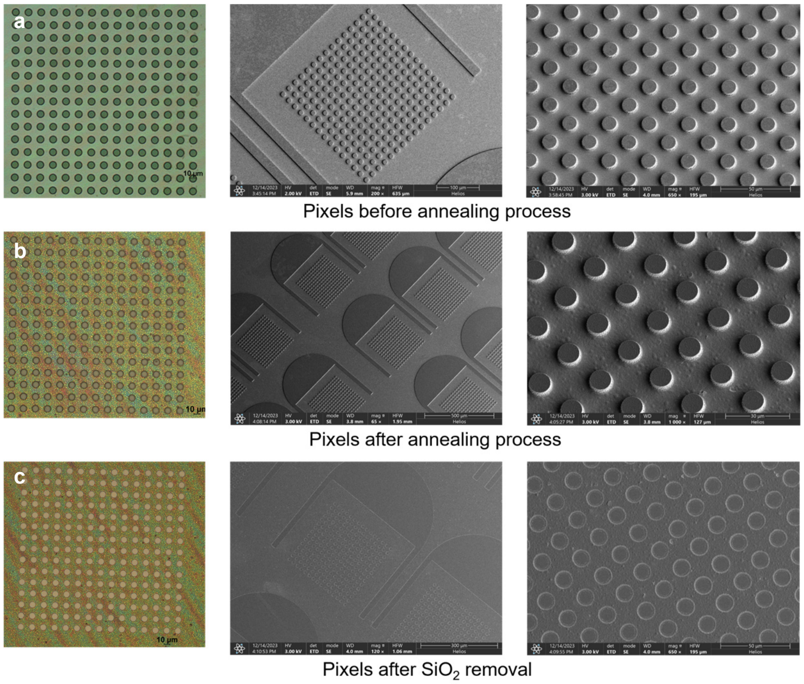

Fig. S2. Optical microscopy and SEM images of pixels (a) before annealing, (b) after annealing, and (c) after SiO_2 removal.

Section SM2:

In the STO method proposed in this work for fabricating micro-LEDs, the removal of the SiO_2 mask must be highly selective to the oxide layer to avoid the risk of leakage or even short circuits. To demonstrate the good selectivity of HF vapor between them, we conducted the measurement of the height difference before and after the removal of SiO_2 using a DEKTAK XT-profile meter, as shown in Fig. S3a to Fig. S3c.

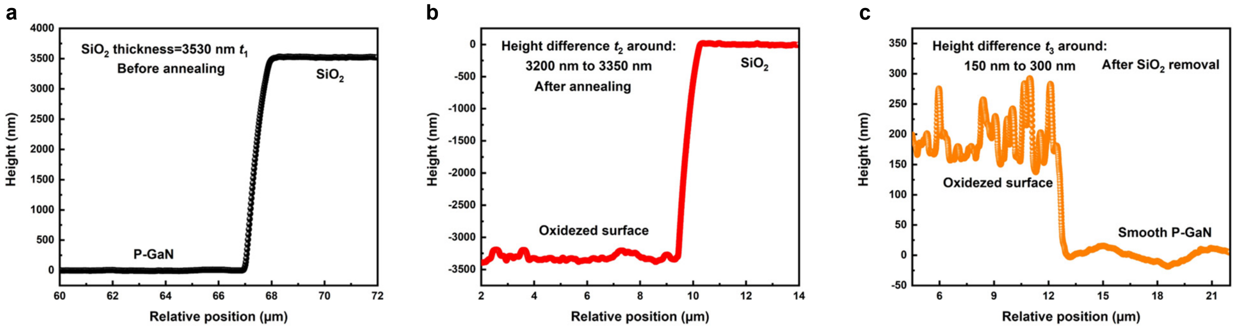

Fig. S3. Profile measurement of pixels (a) before annealing, (b) after annealing, and (c) after SiO_2 removal.

In the test, the thickness of patterned SiO_2 was approximately t_1=3530 nm. After thermal annealing, the height difference between the oxide layer plane and the SiO_2 plane was reduced to a range of approximately t_2=3200 to 3350 nm. The height increase of the oxide plane during oxidation is attributed to thermal expansion and the formation of internal porous. After completely removing all SiO_2 using HF vapor, the oxide plane is still approximately 150 to 300 nm higher than the smooth wafer surface (t_3). The thickness of the oxide layer lost during the HF vapor etching process is given by $t=t_1-t_2-t_3$. Due to the highly roughened surface after oxidation and the limited resolution of the profilometer, we cannot provide a precise etching rate of the oxide layer in HF vapor. However, it is evident that compared to the 3530 nm SiO_2, the value of t is extremely small, providing strong evidence for the high selectivity of the SiO_2 removal process.

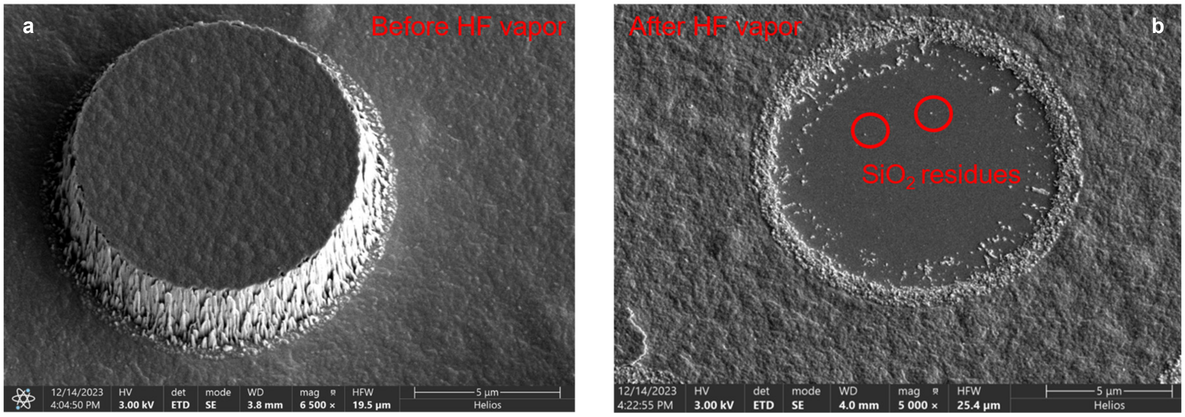

Fig. S4. SEM images of the pixel (a) before and (b) after SiO_2 removal after the STO.

In Fig. S4a and Fig. S4b, we present SEM images to show the changes in pixels before and after HF vapor etching. It can be observed that the oxide layer maintains a similar surface morphology to before etching. The oxide layer plane is slightly higher than the pixel plane, enveloping it, consistent with our profilometer measurements above. Some residual SiO_2 particles are still found within the pixel, indicating that additional etching time with HF vapor and ultrasonic cleaning may be necessary to completely remove them and minimize their impact on device resistance. The edges of the pixels after SiO_2 removal are quite rough, possibly related to the inherent roughness of the SiO_2 mask itself before annealing. In this work, we utilized a dry etching process for SiO_2 patterning. Equipment conditions fluctuating such as chamber environment

and plasma source may lead the variation of sidewall morphology. Fig. S5a and Fig. S5b show SEM images of a reference sample. The etched SiO_2 sidewalls are smoother and steeper, resulting in smoother edges of the pixels after annealing and HF etching. However, we believe that these changes from process condition variations and do not significantly impact the methodology and conclusions of this work.

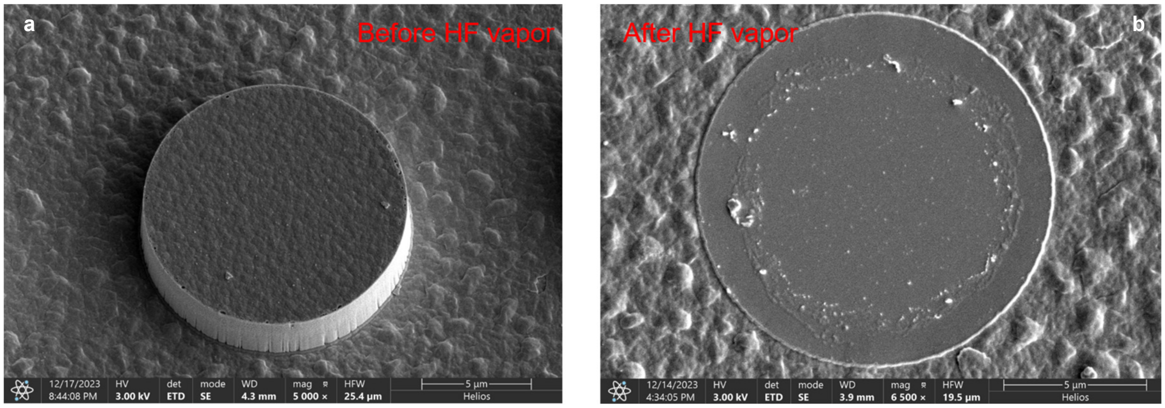

Fig. S5. SEM images of the pixel (a) before SiO_2 removal and (b) after SiO_2 removal after STO (reference sample).

Furthermore, the low leakage current in our device shown in the manuscript can indirectly demonstrate the etching selectivity of HF vapor to SiO_2 and the oxide layer. This is because once the oxide layer is significantly depleted, it will expose the pixel sidewalls, leading to leakage and non-radiative recombination. If the oxide layer is etched further, it may directly result in a short circuit between the n and p electrodes. Clearly, these issues did not occur in our experiments.

Section SM3:

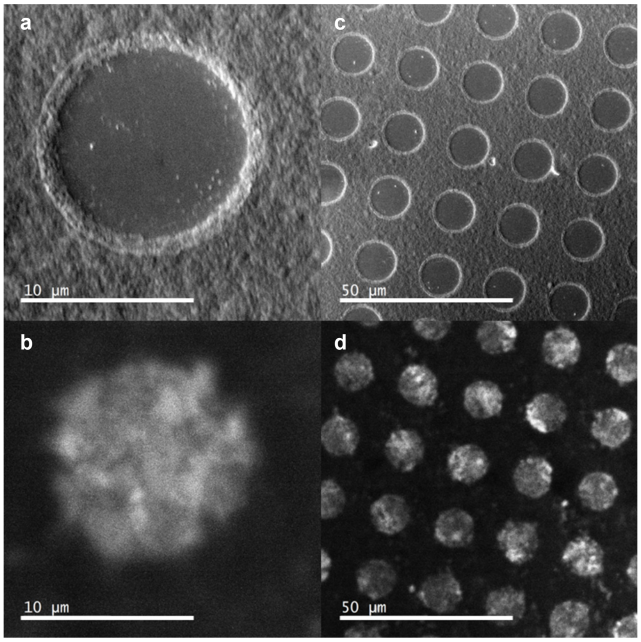

Fig. S6. (a), (c) SEM and (b), (d) CL images of pixels after SiO_2 removal by the HF vapor.

The cathodoluminescence (CL) images measured by the Zeiss Merlin system shown in Fig. S6a to Fig. S6d suggest the emission from pixels is generally uniform, and any observed non-uniformities may stem from epitaxial growth, the STO process, and other fabrication processes. The CL measurement condition: wavelength 560 nm, band pass 50 nm, voltage: 15 KV, current 500 pA.

Section SM4:

Under SiO_2 protection, the LED structure remained intact, exhibiting a clear superlattice structure in Fig. S7a and Fig. S7b. However, once the SiO_2 protection was lost, most of the p-layer and MQW were oxidized and show the distinctly polycrystalline nature of the material formed by oxidation, with multiple crystal orientations mixed together in Fig. S7c and Fig. S7d.

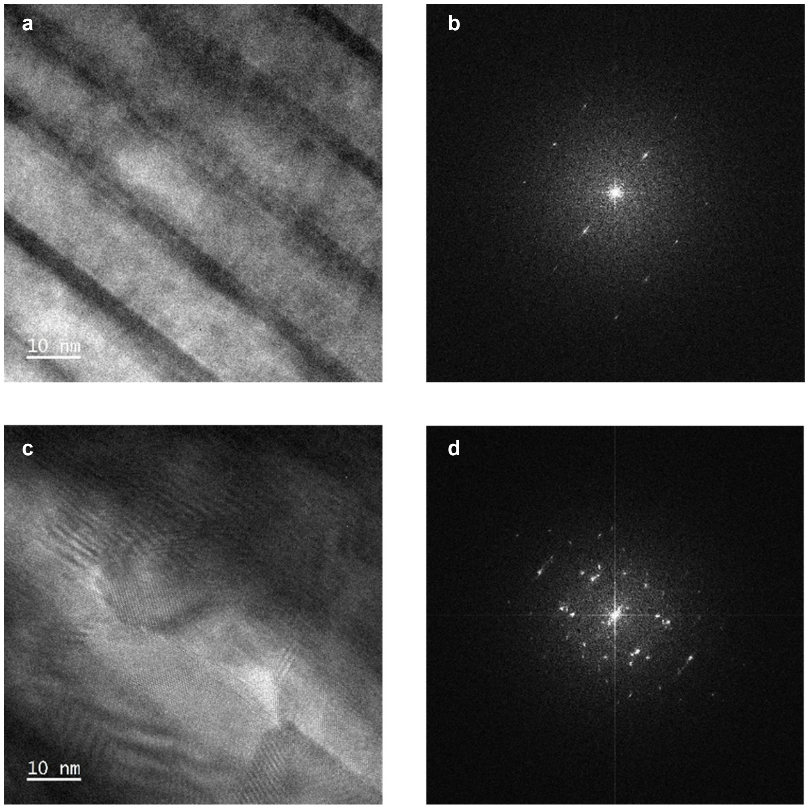

Fig. S7. (a) Magnified TEM image of the LED MQW structure under SiO_2 protection, (b) Fast Fourier Transform (FFT) images of MQW, (c) magnified TEM image of the oxidized material without SiO_2 protection, (d) FFT image of oxide formed by the thermal annealing.

Section SM5:

We cut pixels (different from the one for TEM in the manuscript) using laser cutting and mechanical cracking along the patterned SiO_2 to observe their cross-sections by the Helios SEM system, which was after the STO process but without suffering from the HF vapor etching. The results in Fig. S8 show that, in some pixels, we observed crack propagation and lateral oxidation. This suggests that the cracking phenomenon in the main manuscript is not an isolated case. The crack may result from the thermal mismatch between the oxide and nitride materials during the heating and cooling stages of the STO process.

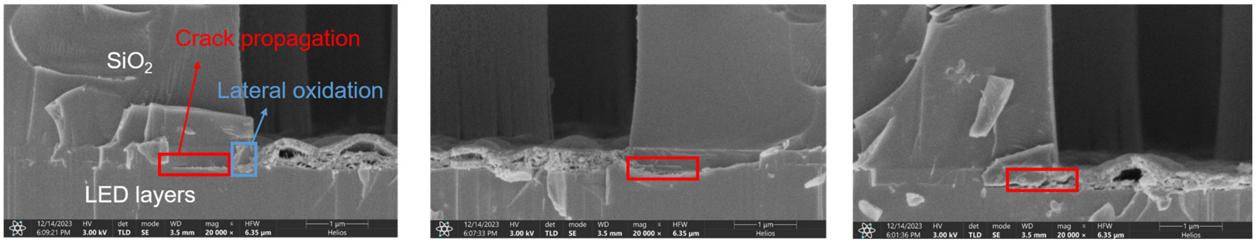

Fig. S8. Cross-section SEM images of the pixel protected by SiO_2 after the STO process (with cracking)

Section SM6:

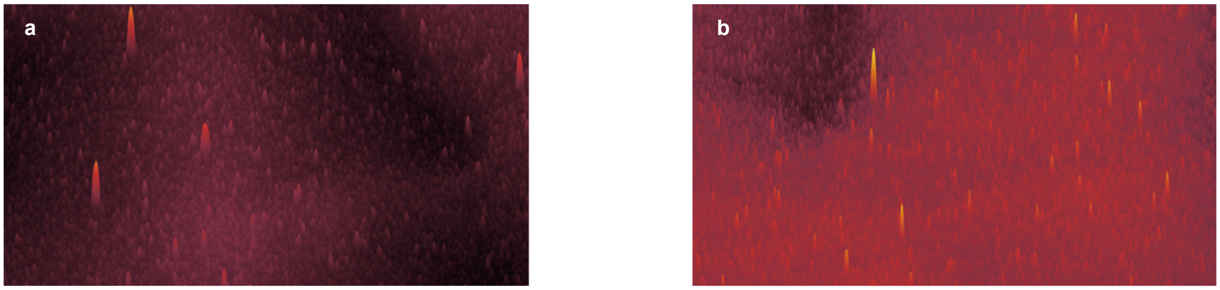

Fig. S9. Enlarged 3D AFM surface image of (a) 4-hour thermal oxidization with 3.5-μm SiO_2 protection and SiO_2 removed by HF vapor, and (b) 8-hour thermal oxidization with 3.5-μm SiO_2 protection and SiO_2 removed by HF vapor.

Compared to the non-annealed reference sample, samples annealed for 4 and 8 hours still maintained a relatively smooth surface overall, consistent with the information in the previous SEM images. However, locally, we observed that the annealed samples exhibited numerous protrusions on the surface, and this phenomenon was slightly more pronounced in the sample annealed for 8 hours as shown in Fig. S9a and Fig. S9b. We believe this was due to oxygen diffusion from SiO_2 into the LED, resulting in surface slight oxidation of the surface and a minor increase in surface roughness.

Section SM7:

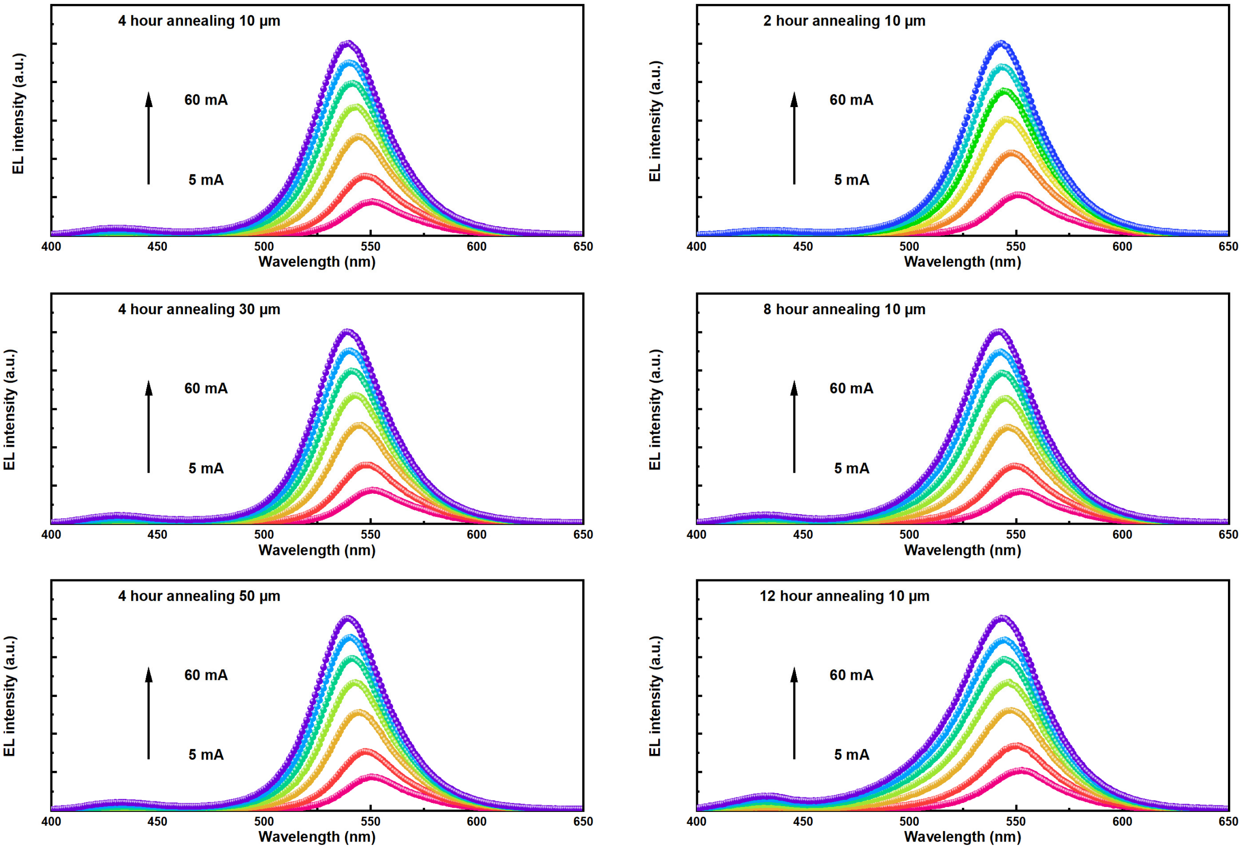

Fig. S10. EL spectrums for all samples mentioned in the manuscript.

Section SM8:

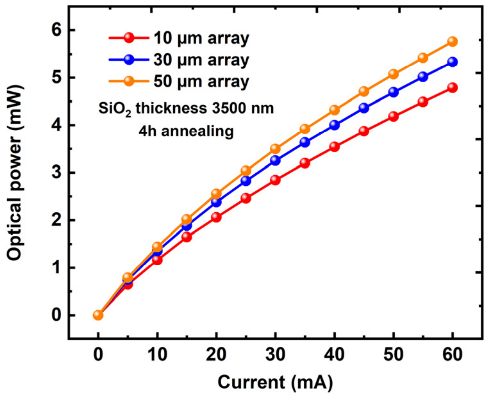

Fig. S11. The light output power for 4 hour-annealed samples with different pixel sizes.

Section SM9:

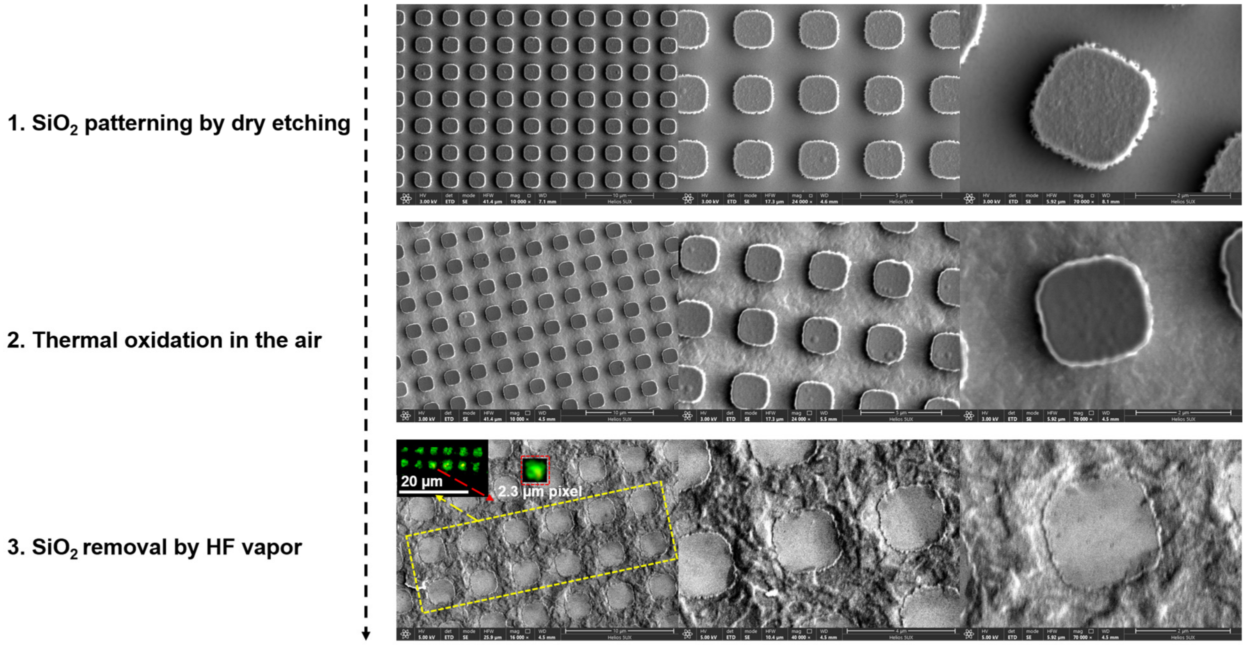

Fig. S12. Fabrication process and corresponding SEM images of STO for 2.3-μm micro-LED array fabrication. The inset image is the EL emission of 2.3-μm micro-LED pixels.

Section SM10:

In this work, we employed SiO_2 as a mask to prevent oxidation and damage to micro-LED pixels during the thermal annealing process. However, oxygen diffusion exhibits isotropy, meaning oxygen atoms can also diffuse laterally beneath the SiO_2 protection. Therefore, we believe that the lateral diffusion rate of oxygen and its impact on the device play a crucial role in determining whether higher pixel density can be achieved. Based on this, we have supplemented a design strategy to achieve a higher resolution micro-LED array shown in Figs. S13 and S14.

In Fig. S13, "*L*" represents the layout line width, "*S*" is the layout space width, and "*d*" is the lateral oxidation length beneath the SiO_2 protection. Due to lateral oxidation, the actual pixel line width becomes

$L' = L - 2d$, and the actual space width becomes $S' = S + 2d$. According to this definition, the actual pixel size L' can be reduced to any dimension until $L \leqslant 2d$ ($L' \geqslant 0$). In a special design, If the same line and space width is desired (i.e., $L' = S'$), then $L - 2d = S + 2d$, and $L = 4d + S$. When $S = 0$, L reaches its minimum value of $4d$, and $L' = S' = L - 2d = 2d$. This situation is shown in Fig. S14.

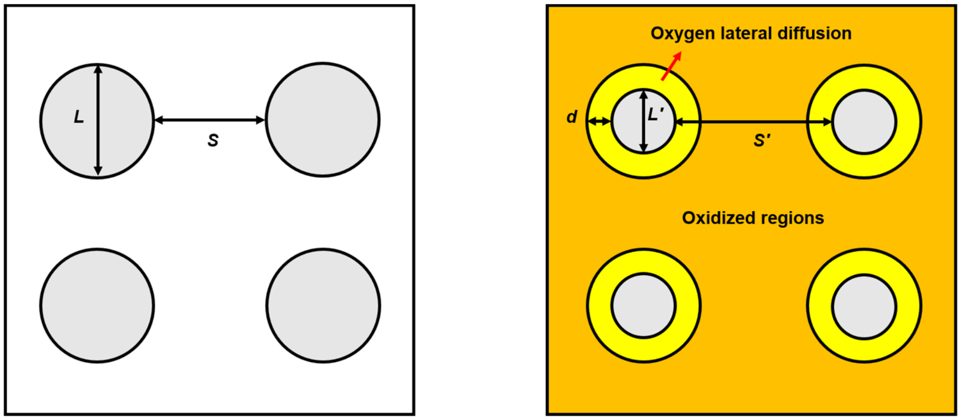

Layout pixels and spaces before annealing

Practical pixels and spaces after annealing

$$\text{Practical } Line/Space = \frac{L}{S'} = \frac{L - 2d}{S + 2d}$$

Fig. S13. A design strategy to achieve a higher resolution micro-LED array.

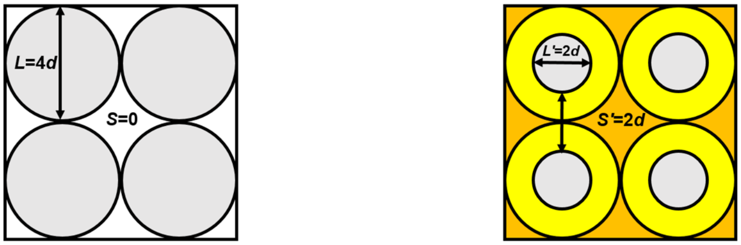

Limitation size for L'=S' before annealing

Limitation size for equal L'=S' after annealing

Fig. S14. A design strategy to achieve a limitation size when L'=S'.

From the TEM image measured in this work, d is approximately 300 nm. This means that, under the described model, we can achieve micro-LED designs with minimum L'/S' = 600 nm/600 nm.

Indeed, achieving such small-dimension pixels involves complex factors, requiring precise control of experimental conditions such as annealing time and ambient conditions to control the crack formation and ensure uniformity and yield.

Section SM11:

In this work, we chose to fabricate arrays rather than individual micro-LEDs because arrays can better reflect the average performance of pixels such as leakage current density and efficiency. In fact, the exact same process can be applied to the fabrication of individual micro-LEDs, as depicted in Fig. S15. The only difference from array manufacturing is that each pixel has its own n-electrode and p-electrode, no longer shared among all pixels in the array.

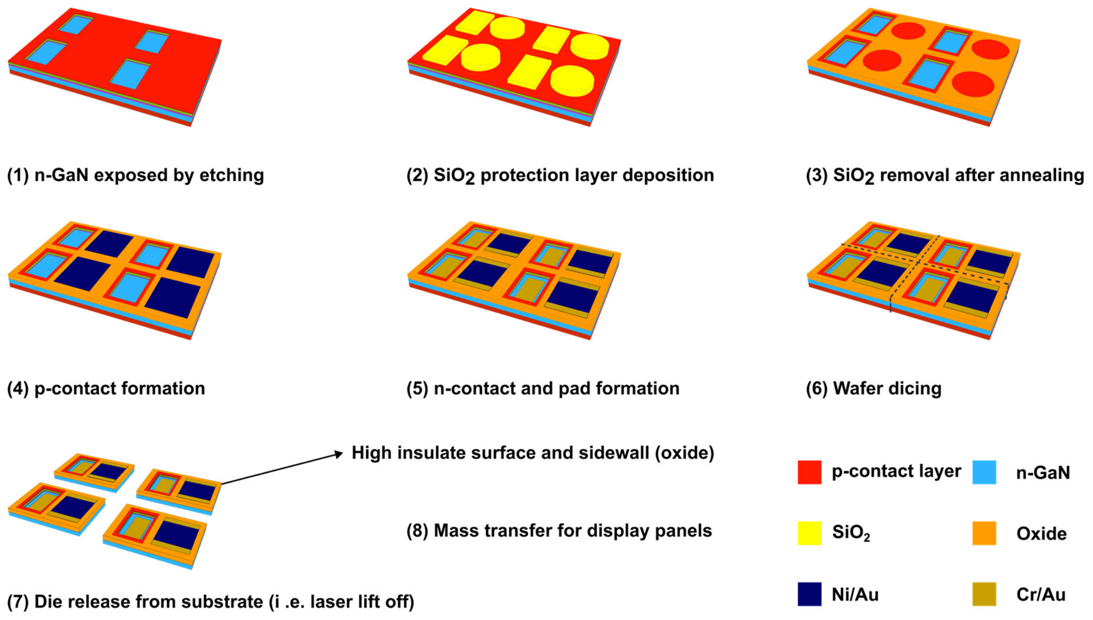

Fig. S15. Individual mesa fabrication and its mass transfer for display applications.

Unlike traditional etching processes, we did not selectively remove the active regions in our approach; instead, we oxidized them into oxides. However, this does not affect the subsequent mass transfer processes for micro-LED pixels. Flip-chip processes, wafer dicing, substrate removal, and pixel transfer can all continue to be applied to our micro-LEDs. Since the periphery of our pixels is entirely composed of high-resistance oxides, processes such as wafer dicing, and pixel bonding to control circuits (electrodes) will not lead to short-circuit or pixel damage leakage. Based on our understanding, we believe that the micro-LED fabrication method we proposed, based on STO, is compatible with mainstream micro-LED mass transfer technologies.
